# Data on plasma levels of apolipoprotein E, correlations with lipids and lipoproteins stratified by *APOE* genotype, and risk of ischemic heart disease

**DOI:** 10.1016/j.dib.2016.01.060

**Published:** 2016-02-05

**Authors:** Katrine L. Rasmussen, Anne Tybjærg-Hansen, Børge G. Nordestgaard, Ruth Frikke-Schmidt

**Affiliations:** a Department of Clinical Biochemistry, Rigshospitalet, Blegdamsvej 9, DK-2100 Copenhagen, Denmark; b The Copenhagen City Heart Study, Frederiksberg Hospital, Nordre Fasanvej 57, DK-2000 Frederiksberg, Denmark; c The Copenhagen General Population Study, Herlev and Gentofte Hospital, Herlev Ringvej 75, DK-2730 Herlev, Denmark; d Department of Clinical Biochemistry, Herlev and Gentofte Hospital, Herlev Ringvej 75, DK-2730 Herlev, Denmark; e Copenhagen University Hospital and Faculty of Health and Medical Sciences, University of Copenhagen, Blegdamsvej 3, DK-2200 Copenhagen, Denmark

## Abstract

Data on correlations of plasma apoE with levels of lipids and lipoproteins stratified by *APOE* genotypes as well as data exploring the association between plasma levels of apoE and risk of ischemic heart disease (IHD) are wanted.

The present data on 91,695 individuals from the general population provides correlations between plasma levels of apoE and lipids and lipoproteins for the three *APOE* genotypes ε33, ε44 and ε22, representing each of the three apoE isoforms. Further, data on extreme groups of plasma apoE (highest 5%) versus lower levels of apoE at enrollment explores risk of IHD and myocardial infarction (MI) and is given as hazard ratios. In addition, IHD and MI as a function of apoE/high-density lipoprotein (HDL) cholesterol ratio, as well as data on lipids, lipoproteins and apolipoproteins are given as hazard ratios. Data is stratified by gender and presented for the Copenhagen General Population Study and the Copenhagen City Heart Study combined.

**Specifications Table**

**Table:** 

Subject area	*Clinical Research – Epidemiology, Biomarkers, Nutrition*
More specific subject area	*Epidemiology, genetics, biomarkers, ischemic heart disease, myocardial infarction, apolipoprotein E, APOE, triglycerides, HDL.*
Type of data	*Table, Figures.*
How data was acquired	*The Copenhagen General Population Study and the Copenhagen City Heart Study are two large prospective studies of the general population.*
Data format	*Analyzed data.*
Experimental factors	*Data on participants in two similar studies of the Danish general population: The Copenhagen General Population Study and The Copenhagen City Heart Study, with altogether 91,695 participants, of whom 4642 developed IHD.*
Experimental features	*Data was obtained from a questionnaire, a physical examination, and from blood samples including DNA extraction. Plasma levels of apolipoprotein E, APOE genotypes as well as lipids and lipoproteins were measured.*
Data source location	*Copenhagen, Denmark.*
Data accessibility	*Data is within this article.*

**Value of the data**

• Data on correlations between plasma levels of apolipoprotein E (apoE) and lipids and lipoproteins for the three *APOE* genotypes, ε33, ε44 and ε22, provides value as isoform specific references.
• These robust human isoform specific data may stimulate experimental research on structure–function relationships.
• The data suggests that triglyceride-mediated pathways may explain the associations between plasma levels of apoE and ischemic heart disease (IHD), and may stimulate the scientific society to explore triglyceride metabolism further.

## 1 Data

**Fig. 1:**
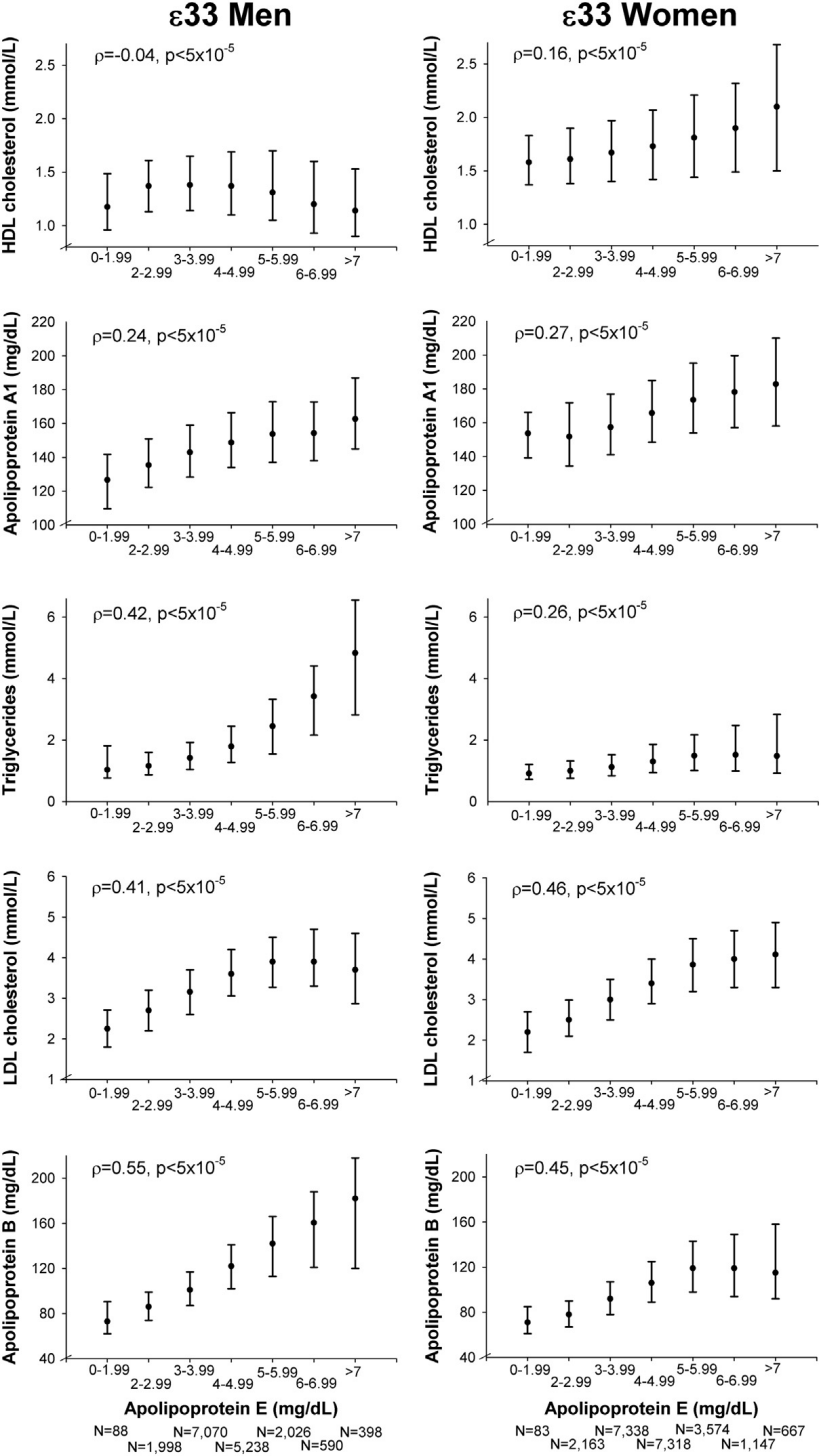
Correlations of lipids, lipoproteins, and apolipoproteins with plasma levels of apolipoprotein E in ε33 individuals in men (left panel) and women (right panel). Values are median and interquartile range. *ρ*=Spearman׳s rho correlation coefficient. *p*=probability values for Spearman׳s rank correlation coefficient for the continuous values of lipids, lipoproteins, and apolipoproteins. ε33=*APOE* ε33 genotype.

**Fig. 2:**
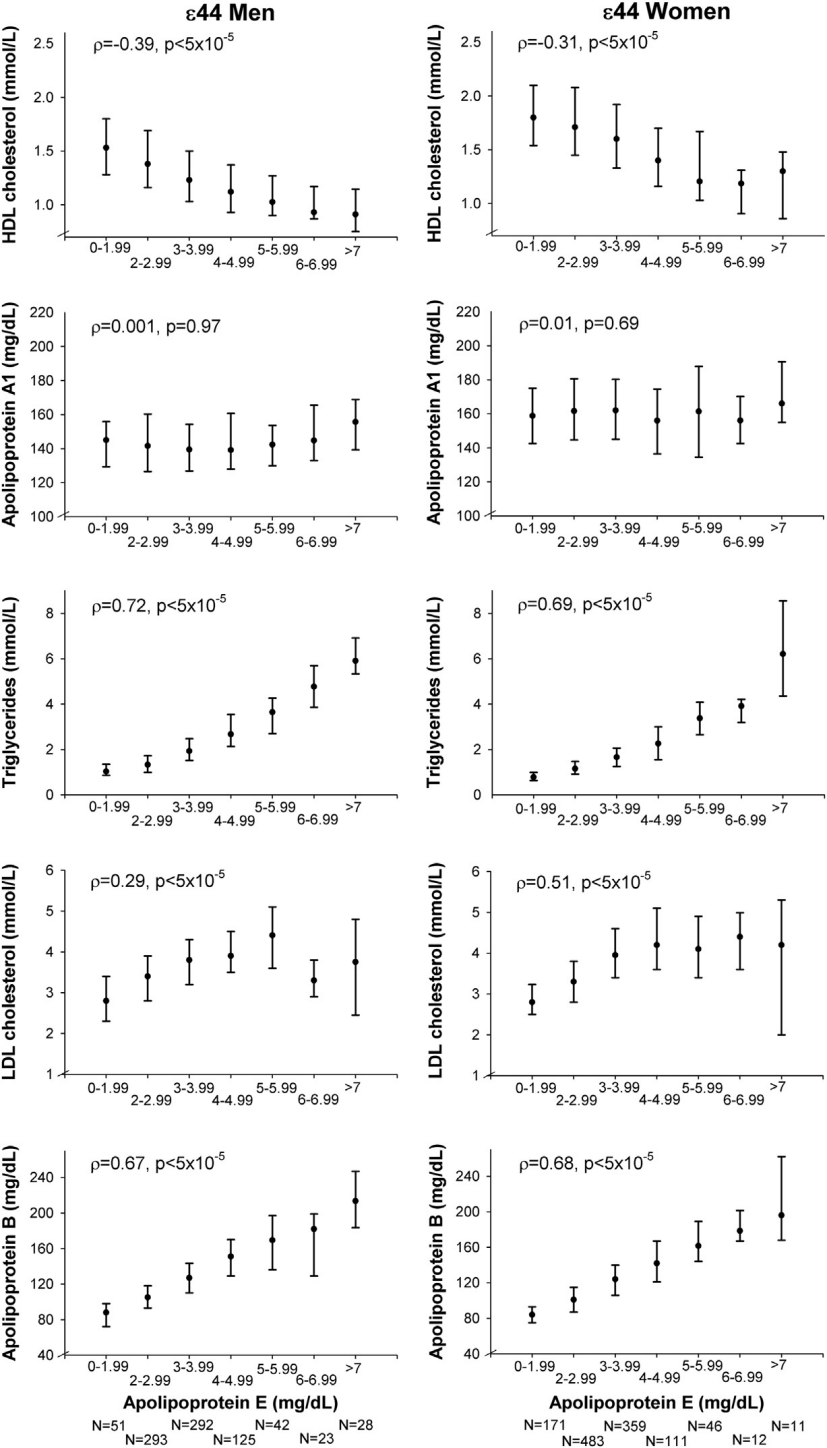
Correlations of lipids, lipoproteins, and apolipoproteins with plasma levels of apolipoprotein E in ε44 individuals in men (left panel) and women (right panel). Values are median and interquartile ranges. *ρ*=Spearman׳s rho correlation coefficient. *p*=probability values for Spearman׳s rank correlation coefficient for the continuous values of lipids, lipoproteins, and apolipoproteins. ε44=*APOE* ε44 genotype.

**Fig. 3:**
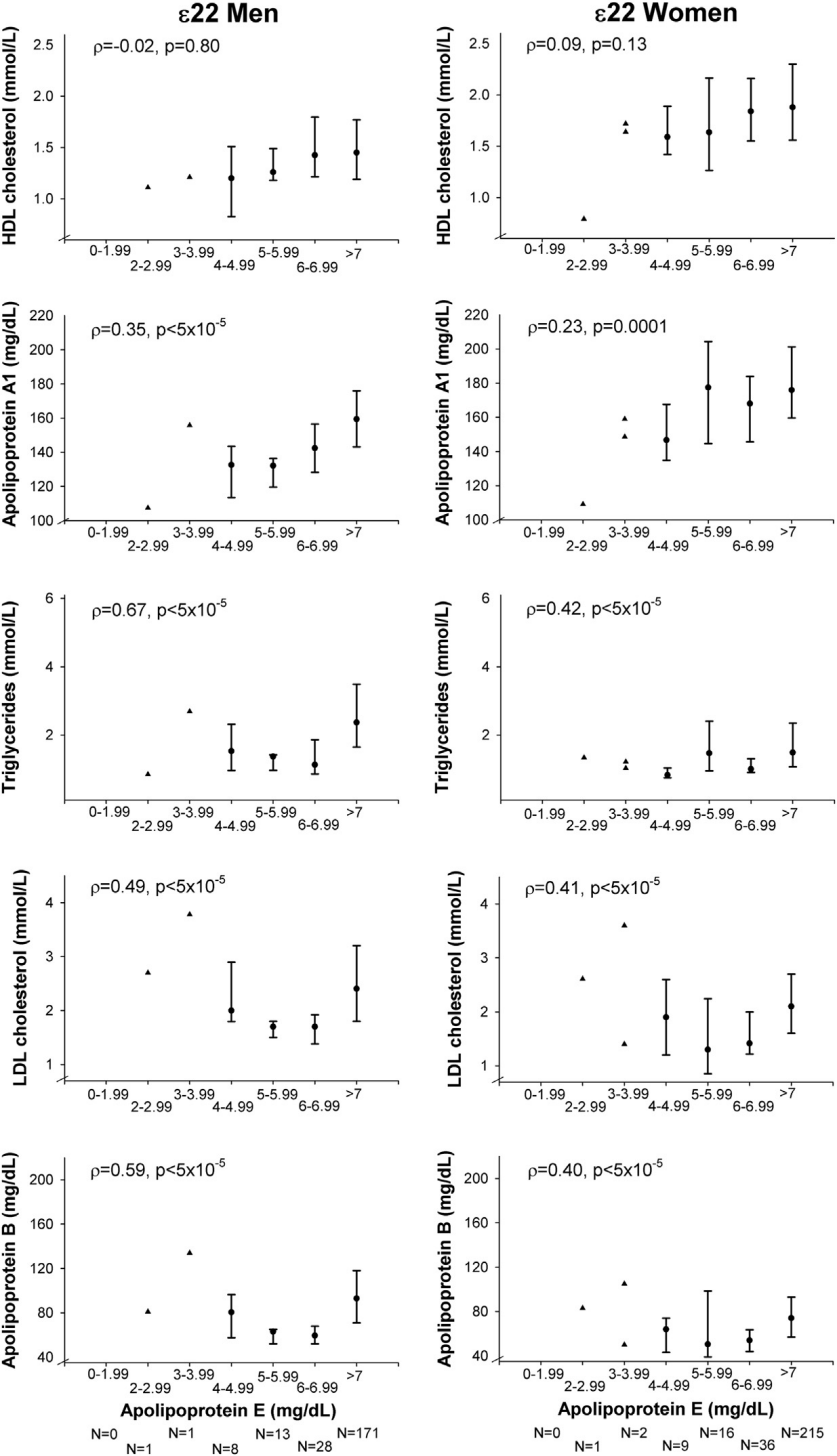
Correlations of lipids, lipoproteins, and apolipoproteins with plasma levels of apolipoprotein E in ε22 individuals in men (left panel) and women (right panel). Values are median and interquartile range. For groups with *N*≤2 values are given for each individual separately (▲). *ρ*=Spearman׳s rho correlation coefficient. *p*=probability values for Spearman׳s rank correlation coefficient for the continuous values of lipids, lipoproteins, and apolipoproteins. ε22=*APOE* ε22 genotype.

**Fig. 4:**
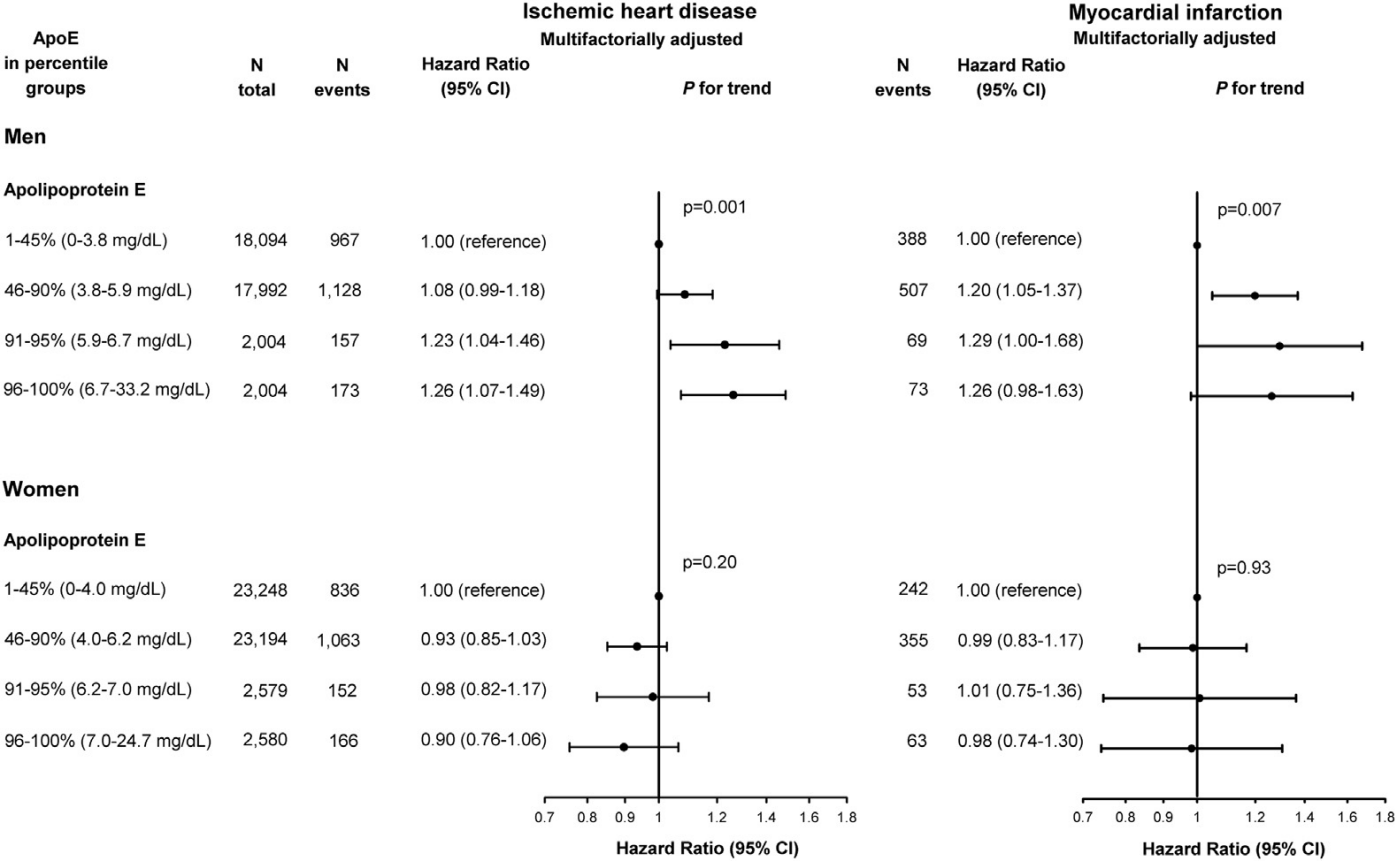
Risk of ischemic heart disease (two left panels) and myocardial infarction (two right panels) as a function of plasma levels of apolipoprotein E in extreme groups in men (upper panels) and women (lower panels). Hazard ratios were multifactorially adjusted for age, body mass index, hypertension, diabetes mellitus, smoking, alcohol consumption, physical inactivity, menopausal status and hormonal replacement therapy (only women), lipid-lowering therapy, and education, and were stratified by sex. apoE=apolipoprotein. CI=confidence interval.

**Fig. 5:**
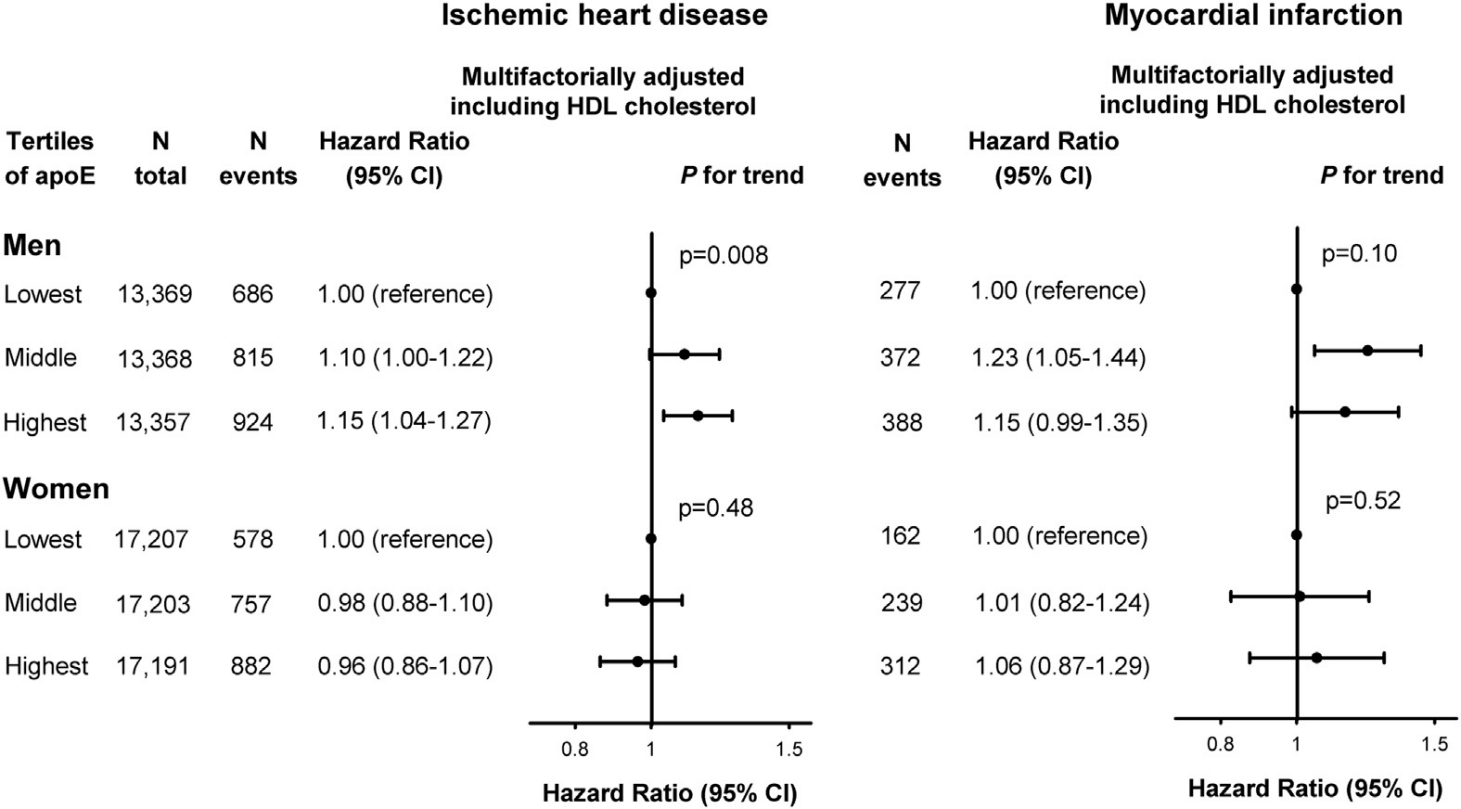
Risk of ischemic heart disease (left panel) and myocardial infarction (right panel) as a function of plasma levels of apolipoprotein E in tertiles, in men (upper panel) and women (lower panel) with adjustment including HDL cholesterol. Hazard ratios were multifactorially adjusted for age, body mass index, hypertension, diabetes mellitus, smoking, alcohol consumption, physical inactivity, menopausal status and hormonal replacement therapy (only women), lipid-lowering therapy, education, and HDL cholesterol, and were stratified by sex. Tertiles of HDL cholesterol were used for adjustment for HDL cholesterol. We tested highest and middle versus lowest tertile of apoE. CI=confidence interval. HDL=high-density lipoprotein.

**Fig. 6:**
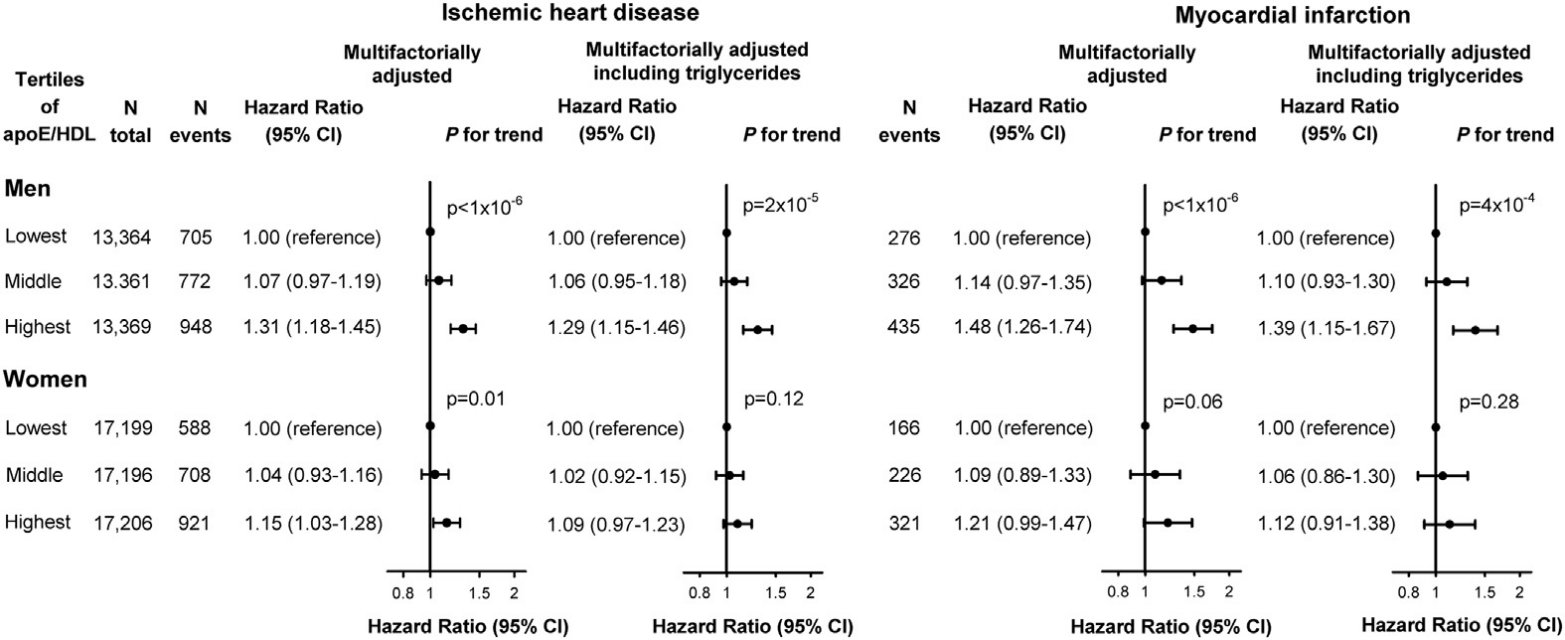
Risk of ischemic heart disease (two left panels) and myocardial infarction (two right panels) as a function of apoE/HDL cholesterol in tertiles, in men (upper panel) and women (lower panel). Hazard ratios were multifactorially adjusted for age, body mass index, hypertension, diabetes mellitus, smoking, alcohol consumption, physical inactivity, menopausal status and hormonal replacement therapy (only women), lipid-lowering therapy, and education, and were stratified by sex. Further adjustment included triglycerides in tertiles. apoE/HDLcholesterol=ratio of apolipoprotein E relative to high-density lipoprotein cholesterol. We tested highest and middle versus lowest tertile. CI=confidence interval.

**Fig. 7:**
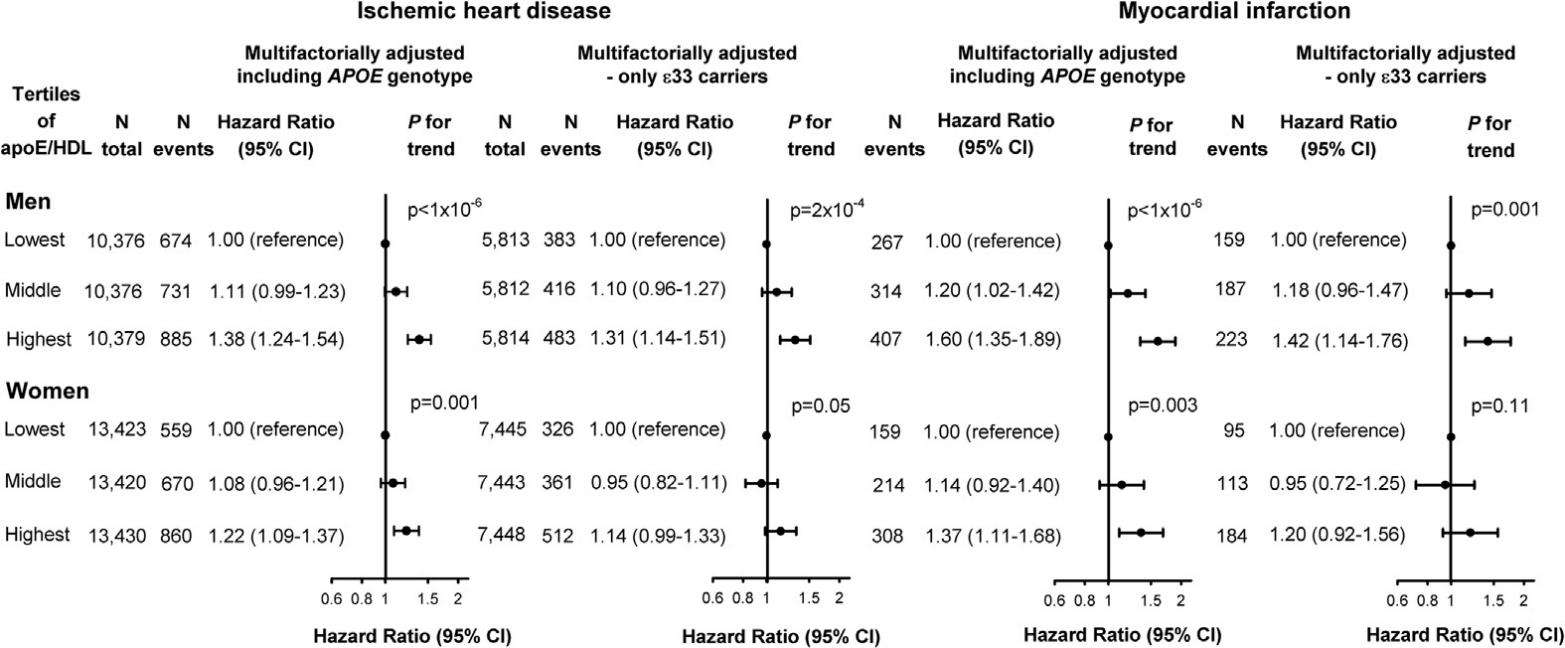
Risk of ischemic heart disease (two left panels) and myocardial infarction (two right panels) as a function of apoE/HDL cholesterol in tertiles, in men (upper panel) and women (lower panel) in *APOE* genotype adjusted and ε33 stratified analyses. Hazard ratios were multifactorially adjusted for age, body mass index, hypertension, diabetes mellitus, smoking, alcohol consumption, physical inactivity, menopausal status and hormonal replacement therapy (only women), lipid-lowering therapy, and education, and were stratified by sex. Analyses were further adjusted for *APOE* genotype or analyzed in individuals with ε33 *APOE* genotype only. We tested highest and middle versus lowest tertile. apoE/HDL cholesterol=ratio of apolipoprotein E relative to high-density lipoprotein cholesterol. CI=confidence interval. ε33=*APOE* ε33 genotype.

**Fig. 8:**
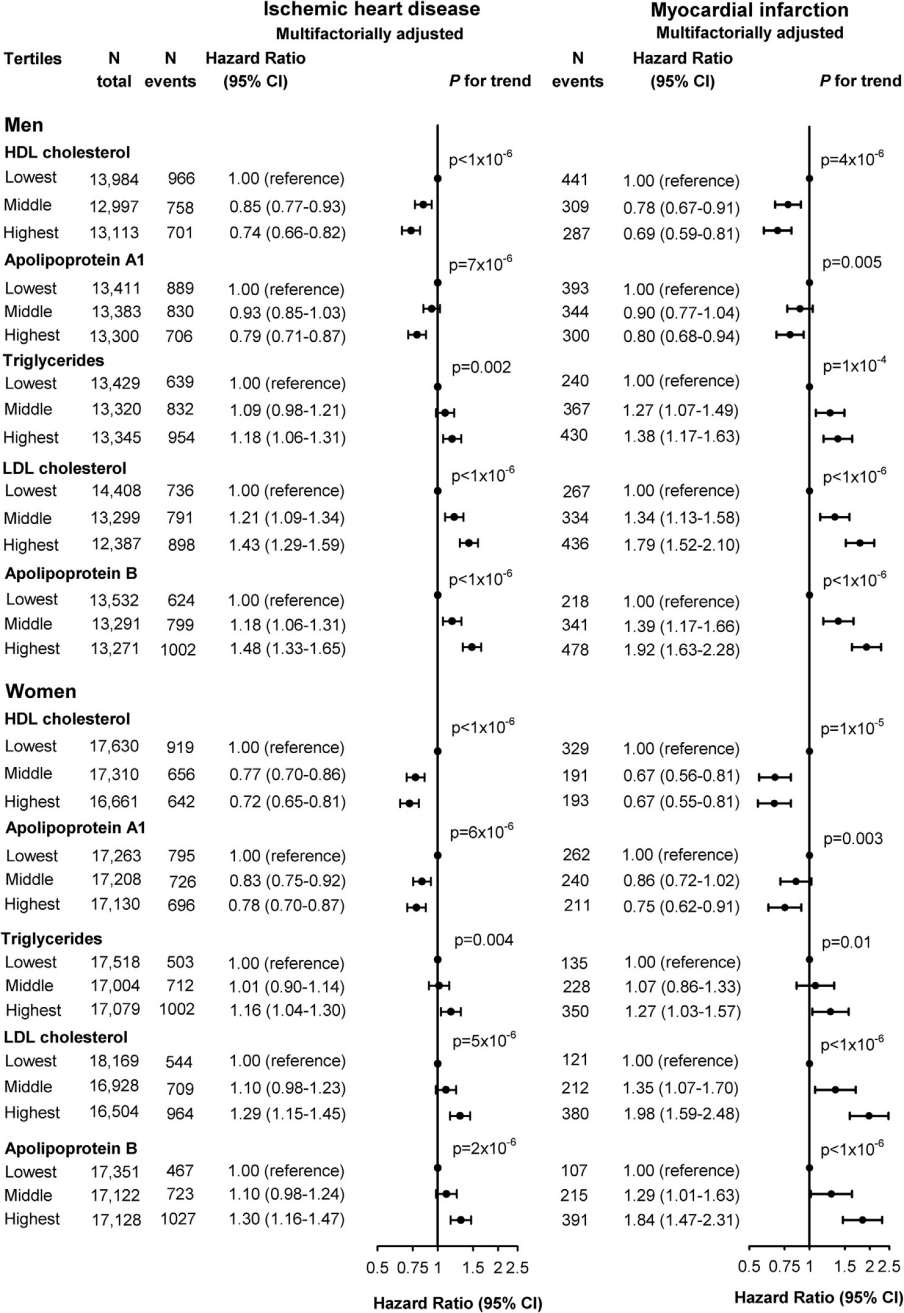
Risk of ischemic heart disease (left panel) and myocardial infarction (right panel) as a function of lipids, lipoproteins, and apolipoproteins. Hazard ratios were multifactorially adjusted for age, body mass index, hypertension, diabetes mellitus, smoking, alcohol consumption, physical inactivity, menopausal status and hormonal replacement therapy (only women), lipid-lowering therapy, and education, and were stratified by sex. HDL=high-density lipoprotein. LDL=low-density lipoprotein. CI=confidence interval.

The present data in 91,695 individuals from the general population provides correlations between plasma levels of apoE and lipids and lipoproteins for the three *APOE* genotypes ε33, ε44 and ε22, representing each of the three apoE isoforms. Further, data in the form of hazard ratios for extreme groups of plasma apoE (highest 5%), tertiles of apoE, tertiles of apoE/HDL cholesterol ratio as well as tertiles of lipids, lipoproteins and apolipoproteins for risk of IHD and myocardial infarction (MI) is given. Additionally, data on characteristics of participants by apoE tertile is presented. Data is given as analyzed data (Fig. 1, Fig. 2, Fig. 3, Fig. 4, Fig. 5, Fig. 6, Fig. 7, Fig. 8 and Table 1).

## 2 Experimental design, materials and methods

We used data from two similar studies of the Danish general population, the Copenhagen General Population Study (CGPS) and the Copenhagen City Heart Study (CCHS), with altogether 91,695 participants, of whom 4642 developed IHD [1], [2], [3]. Data was obtained from a questionnaire, a physical examination, and from blood samples including DNA extraction [1], [2], [3]. The CGPS is a prospective study of the Danish general population initiated in 2003 with ongoing enrollment, whereas the CCHS is a prospective study of the Danish general population initiated in 1976–78 with follow-up examinations in 1981–83, 1991–94, and 2001–03. Studies were approved by institutional review boards and Danish ethical committees, and were conducted according to the Declaration of Helsinki. Written informed consent was obtained from participants. All participants were white and of Danish descent. There was no overlap of individuals between the CGPS and the CCHS.

Information on diagnoses of IHD (World Health Organization International Classification of Diseases (ICD), 8th version, ICD8: 410-414, 10th version ICD10:I20-I25) was collected from the National Danish Patient Registry and the National Danish Causes of Death Registry. IHD was ICD8 410-414; and ICD10 I20-I25; MI constitutes a subgroup (ICD8 410 and ICD10 I21-I22).

Follow-up time began at the time of blood sampling (2003 and onwards for CGPS and 1991–94 or 2001–03 for CCHS). Follow-up ended at occurrence of event, death, emigration, or on April 10th, 2013 (last update of the registry), whichever came first. Median follow-up was 5 years (range 0–22 years) with no individuals lost to follow-up.

Biochemical and genetic analyses were similar to analyses provided by Rasmussen et al. [4], [5].

For the statistical analyses we used Stata/S.E. version 12.0 (Stata Corp., College Station, Texas, USA). *P*-values<0.0001 are given as powers of 10. Student׳s *t*-test and Pearson׳s *χ*^2^ -test were used in comparisons of continuous and categorical variables, respectively. Spearman׳s rank correlation was used for the correlation of continuous values of lipids, lipoproteins, and apolipoproteins with continuous values of apoE, stratified by the homozygote *APOE* genotypes, ε33, ε44 and ε22, representing each of the three apoE isoforms. Missing data on categorical and continuous covariates (<1.0%) were imputed from age, sex and population using multiple imputation with ten imputations. Multinomial logistic regression was applied for categorical variables and linear regression for continuous variables, and was performed using the “mi impute mlogit” and “mi impute chained (regress)” commands in Stata.

Cox proportional hazards regression models estimated hazard ratios for MI and IHD as a function of extreme groups and tertiles of plasma levels of apoE, tertiles of apoE/HDL cholesterol ratios, and tertiles of lipids, lipoproteins, and apolipoproteins. In the Cox models we adjusted for age (automatic adjustment as age is the time scale) and known risk factors as detailed in legend to Table 1. Further adjustment included adjustment for triglycerides in tertiles, HDL cholesterol in tertiles or *APOE* genotype. Further ε33 stratified analyses for apoE/HDL cholesterol ratios in tertiles were performed. As gender and apoE levels interacted in predicting IHD (*p*=0.04), the analyses were performed separately for each gender, and data is presented stratified by gender. Data is presented for the CGPS and the CCHS combined.

## Figures and Tables

**Table 1:** Characteristics of participants by apolipoprotein E tertile.

	Men			Women		
	Lowest tertile	Middle tertile	Highest tertile	Lowest tertile	Middle tertile	Highest tertile
No. of individuals (%)	13,369 (33)	13,368 (33)	13,357 (33)	17,207 (33)	17,203 (33)	17,191 (33)
Age (years)	55.9±0.1	57.2±0.1^*a*^	57.7±0.1^*a*^	51.8±0.1	57.7±0.1^*a*^	60.9±0.09^*a*^
Apolipoprotein E (mg/dL)	3.0±0.004	4.0±0.002^*a*^	5.7±0.01^*a*^	3.1±0.004	4.2±0.002^*a*^	6.0±0.01^*a*^
Total cholesterol (mmol/L)	5.1±0.008	5.7±0.008^*a*^	6.1±0.01^*a*^	5.2±0.007	5.8±0.008^*a*^	6.2±0.009^*a*^
HDL cholesterol (mmol/L)	1.4±0.003	1.42±0.004	1.4±0.004^*a*^	1.7±0.003	1.8±0.004^*a*^	1.9±0.004^*a*^
Triglycerides (mmol/L)^*c*^	1.3±0.01	1.6±0.01^*a*^	2.2±0,02^*a*^	1.1±0.01	1.3±0.01^*a*^	1.5±0.01^*a*^
LDL cholesterol (mmol/L)	3.0±0.007	3.4±0.008^*a*^	3.6±0.009^*a*^	3.0±0.006	3.4±0.007^*a*^	3.6±0.008^*a*^
Apolipoprotein B (mg/dL)	97.3±0.2	114.5±0.2^*a*^	134.1±0.4^*a*^	91.6±0.2	106.0±0.2^*a*^	116.1±0.3^*a*^
Apolipoprotein A1 (mg/dL)	142.9±0.2	148.9±0.2^*a*^	156.2±0.2^*a*^	159.8±0.2	166.8±0.2^*a*^	178.2±0.2^*a*^
Body mass index (kg/m^2^)	25.8±0.03	26.7±0.03^*a*^	27.6±0.03^*a*^	24.5±0.03	25.7±0.03^*a*^	26.5±0.04^*a*^
Hypertension (%)	7809 (58)	8698 (65)	9493 (71)^*a*^	6941 (40)	9267 (54)	10,719 (62)^*a*^
Diabetes mellitus (%)	761 (6)	484 (4)	548 (4)^*a*^	442 (3)	467 (3)	502 (3)
Smoking (%)	3204 (24)	3057 (23)	3146 (24)	3764 (22)	3647 (21)	3432 (20)^*a*^
Alcohol consumption (%)	2438 (18)	2790 (21)	3210 (24)^*a*^	2162 (13)	2593 (15)	2773 (16)^*a*^
Physical inactivity (%)	5585 (42)	6091 (46)	6646 (50)^*a*^	9041 (53)	9557 (56)	9931 (58)^*a*^
Postmenopausal (%)^*d*^	–	–	–	8320 (48)	11842 (69)	13,821 (80)^*a*^
Hormonal replacement therapy (%)^*d*^	–	–	–	1935 (11)	1791 (10)	1768 (10)^*b*^
Lipid-lowering therapy (%)	1604 (12)	1115 (8)	730 (5)^*a*^	1380 (8)	1402 (8)	1074 (6)^*a*^
Education <8 years (%)	1612 (12)	1713 (13)	1796 (13)^*b*^	1370 (8)	2224 (13)	2711 (16)^*a*^

Values are mean (±standard error of the mean) or percent, and are from the day of enrollment (2003 and onwards for the Copenhagen General Population Study and 1991–94 or 2001–03 for the Copenhagen City Heart Study). Missing data on categorical and continuous covariates (<1.0%) were imputed from age and population using multiple imputation. Hypertension was use of anti-hypertensive medication and/or a systolic blood pressure of 140 mm Hg or greater, and/or a diastolic blood pressure of 90 mm Hg or greater. Diabetes mellitus was self-reported disease, use of insulin or oral hypoglycaemic agents, and/or non-fasting plasma glucose levels of more than 11 mmol/L (>198 mg/dL). Smoking was current smoking. Alcohol consumption was >14/21 units per week for women/men (1 unit=12 g alcohol, equivalent to one glass of wine or one beer (33 cL)). Physical inactivity was ≤4 hours per week of light physical activity in leisure time. Women reported menopausal status and use of hormonal replacement therapy. Lipid-lowering therapy was primarily statins (yes/no), and education was <8 years of education ^*a*^*p*<0.001 and ^*b*^*p*<0.05 by Student׳s *t*-test for lowest apolipoprotein E tertile versus middle tertile and for lowest tertile versus highest tertile, respectively, or Pearson׳s *χ*^2^-test for a 2×3 table with the significance level for the overall 2×3 table indicated in the columns with the highest tertile. ^*c*^Geometric mean±standard error of the mean for unimputed triglyceride levels is shown. ^*d*^In women only.
